# Flow Cytometric Evaluation of T Cell Activation Markers after Cardiopulmonary Bypass

**DOI:** 10.1155/2014/801643

**Published:** 2014-02-06

**Authors:** Maja-Theresa Dieterlen, Hartmuth B. Bittner, Attila Tarnok, Jens Garbade, Stefan Dhein, Friedrich W. Mohr, Markus J. Barten

**Affiliations:** ^1^University Hospital Leipzig, Heart Center, Department of Cardiac Surgery, 04289 Leipzig, Germany; ^2^Florida Hospital Orlando, Department of Cadiothoracic Transplantation and Advanced Cardiac Surgery, Orlando, FL 32803, USA; ^3^University Hospital Leipzig, Heart Center, Department of Pediatric Cardiology, 04289 Leipzig, Germany

## Abstract

*Background*. Cardiopulmonary bypass surgery (CPBS) is associated with an increased risk for infections or with subsequent organ dysfunction. As T cell activation is a central mechanism during inflammatory processes, we developed an assay to evaluate T cell activation pathways in patients undergoing CPBS. *Methods*. Blood was obtained from eleven patients undergoing CPBS preoperatively, on postoperative day (POD)-3, and on POD-7 and was stimulated with different concentrations of Concanavalin A (ConA). Cyclosporine and sirolimus, inhibiting different pathways of the T cell cycle, were added to blood ex vivo. Expression of T cell activation markers CD25 and CD95 was analyzed by flow cytometry. *Results*. In untreated blood, expression of CD25 and CD95 significantly increased with higher ConA concentrations (*P* < 0.05) and decreased for all ConA concentrations for both antigens over the study time (*P* < 0.05). Independently from the ConA concentration, inhibition of CD25 and CD95 expression was highest preoperatively for sirolimus and on POD-3 for cyclosporine. At all time points, inhibition of CD25 and CD95 expression was significantly higher after cyclosporine compared to sirolimus treatment (*P* < 0.001). *Conclusion*. Our results showed that different pathways of T cell activation are impaired after CPBS. Such knowledge may offer the opportunity to identify patients at risk for postoperative complications.

## 1. Introduction

Dysfunction of the immune system after cardiopulmonary bypass surgery (CPBS) is a well-known phenomenon and associated with an increased risk for postoperative complications such as infections or pronounced effusions and edema [1]. Despite the benefits of CPBS, this intervention is associated with a significant morbidity in 1-2% of the patients [2], mainly caused by the concomitant inflammatory response.

Complications of CPBS could be linked to either immune activation or immune suppression [3]. Alterations of the specific immune system after CPBS generate a systemic inflammatory response involving activated neutrophils, the disturbance of the balance between proinflammatory and anti-inflammatory cytokines, and interactions with other mediators such as arachidonic acid [4]. At the same time, CPBS also induces the cellular and humoral constituents of the adaptive immune system to undergo quantitative and qualitative changes, leading to a temporary immunodeficiency [5].

Decreases in both number and function of circulating lymphocytes have been reported postoperatively in patients undergoing CPBS [6, 7]. Further studies showed drastic decreases of circulating T cells and reduced levels of T cell-produced cytokines but an increasing percentage of B cells in the postbypass period [3, 8].

In particular the function of Th1 cells seems to be suppressed after CPBS resulting in a shift of the Th1/Th2 ratio towards the latter [9]. An elevated Th2 response was related to increased vascular permeability after CPBS in pediatric patients [1] and possibly even enhanced humoral immune response [10].

Besides immunosuppressive effects immune activating processes seem to be associated with CPBS. The purpose of this study was to further elucidate specific mechanisms of disturbed T cell function after CBPS. We therefore implemented the application of whole blood assays that were used in similarly for pharmacodynamic monitoring, to analyze the expression of specific activation markers of T cell function [11]. The addition of immunosuppressive drugs with different modes of action to whole blood allowed us to investigate the alteration of different T cell functional pathways.

## 2. Materials and Methods

### 2.1. Study Subjects

Blood was obtained from 11 patients undergoing CPBS for open heart procedures at different time points: preoperatively (POD-0), on day 3 (POD-3), and on day 7 (POD-7) postoperatively. Collected blood was stored at room temperature for use within one hour. Surgical procedures performed with CPB included coronary artery bypass grafting (CABG, *n* = 4), aortic valve replacement (AVR, *n* = 3), or combined procedures (AVR and CABG, *n* = 4).

The study was approved by the Ethic Committee of the Medical Faculty of the University Leipzig (number 2004-05-WGK-3). The subjects gave informed consent.

### 2.2. Reagents

Culture medium (CM) was prepared using RPMI 1640 supplemented with 100 U/mL of penicillin, 100 *μ*g/mg streptomycin (both from Sigma, Steinheim, Germany), and 2 mM L-glutamine (Biochrom, Berlin, Germany). Concanavalin A (ConA) (Sigma) was diluted in CM to a concentration of 600 *μ*g/mL and stored at −70°C. Antibodies were purchased from Becton Dickinson (BD, Heidelberg, Germany). Phosphate buffered saline (PBS) was made by dissolving 7.013 g NaCl, 0.2 g KCl, 1.513 g Na_2_HPO4, and 0.2 g KH_2_PO4 (all purchased from Roth) in 1 liter distilled water and by adjusting the pH to 7.4. Red blood cell (RBC) lysing solution was purchased from Becton Dickinson and diluted 1 : 10 in distilled water before use. Washing buffer was obtained from BD Biosciences (San Diego, USA). Formaldehyde solution and absolute methanol was purchased from Merck (Darmstadt, Germany).

### 2.3. Drugs

Cyclosporine A (CsA; MW 1202, Sigma) and sirolimus (SRL; MW 914.2, Sigma) were diluted in absolute ethanol as stock solutions of 1 mM. Drug concentrations were chosen as described previously [12]: SRL: 100 nM, CsA: 1000 nM.

### 2.4. Blood

Ten *μ*L of the drug dilutions (only 5 *μ*L in the case of SRL to minimize any ethanol effect) was added to 390 *μ*L (or 395 *μ*L in the case of SRL) heparinized whole blood to produce the desired concentrations. Drugs were allowed to equilibrate at 37°C for 30 min to ensure homogeneous distribution of all the constituents of the blood.

### 2.5. Whole Blood Mitogen-Stimulated T Cell Assay

Heparinized blood (20 *μ*L) was diluted in CM (175 *μ*L) and added to the wells of flat bottom 24-well tissue culture microtiter plates (Greiner bio-one, Frickenhausen, Germany). To each well, 5 *μ*L of diluted ConA or 5 *μ*L CM (unstimulated cultures) was added to give a final volume of 200 *μ*L. The final concentration of ConA in 20 *μ*L blood culture was 5 *μ*g/mL, 7.5 *μ*g/mL, 10 *μ*g/mL, and 15 *μ*g/mL, respectively. The final dilution of blood in the well was 1/10. All cultures were incubated for 3 days at 37°C in a humidified 5% CO_2_-air water jacketed incubator [13].

### 2.6. Flow Cytometric Analysis of T Cell Activation Markers

Five *μ*L of each antibody (CD3-PerCP, CD25-PE, CD95-FITC) was added to 200 *μ*L blood culture. Unstimulated blood cultures were used as negative controls. Isotype controls included replacement of specific antibodies with isotype mouse immunoglobulins. After vortexing and incubation for 15 min at room temperature in dark, 2 mL lysing solution was added and RBCs were lysed for 8 min at room temperature in dark. Samples were centrifuged (Rotina 35R, Hettich Zentrifugen, Baech, Switzerland) for 5 min at 460 ×g. After pelleting, 2 mL washing solution was added and samples were centrifuged for 5 min. Prior to analysis, leukocytes were resuspended in 300 *μ*L PBS containing 1% (volume/volume) formaldehyde. Samples were analyzed using a FACS Scan Flow Cytometer and *CellQuest* Software (both BD). T cells were identified as described previously [12]. Ten thousand events were analyzed per sample.

### 2.7. Analysis of Data

All data, percentages of expression or percentages of inhibition, were shown as mean ± standard error of the mean (SEM). Drug effects were calculated as described previously [12]:

(1) $$\begin{array}{c} \text{Percent}\;\text{inhibition}=\left[1-\left(\frac{\text{Treatment}}{\text{Pretreatment}}\right)\right]\times 100, \end{array}$$

where “Pretreatment” represents the results obtained from stimulated blood without addition of drugs and “Treatment” represents the results from stimulated blood prepared by adding different concentrations of the drug to whole blood. Statistical significance was analyzed between two groups with equal variance by the one-way ANOVA followed by the Mann and Whitney rank sum test and by the 2-tailed Student's *t*-test. A *P* value of less than 0.05 was considered to be statistically significant. Correlations between mean time on cardiopulmonary bypass, inhibition of expression of T cell markers, and ConA concentration were determined using the Pearson product rank order (ANOVA on ranks).

## 3. Results

### 3.1. Patients Demographics

The mean age was 70.0 ± 2.9 yrs with a balanced ratio of gender (male 45%, female 55%). The mean time of CPBS was 109.0 ± 10.6 min. White blood cell (WBC) count showed a significant increase on POD-3 (10.6 ± 0.8 Gpt/*μ*L; *P* < 0.02) with a consecutive decrease on POD-7 (9.5 ± 0.5 Gpt/*μ*L), however, not declining to preoperative values (POD-0 mean 7.7 ± 0.7 Gpt/*μ*L). Lymphocyte counts did not change significantly after CPBS (POD-3: 2.8 ± 0.5 Gpt/*μ*L; POD-7 2.4 ± 0.3 Gpt/*μ*L) compared to POD-0 (2.2 ± 0.4 Gpt/*μ*L).

### 3.2. Expression of T Cell Activation Markers in Untreated Whole Blood

**Figure 1:**
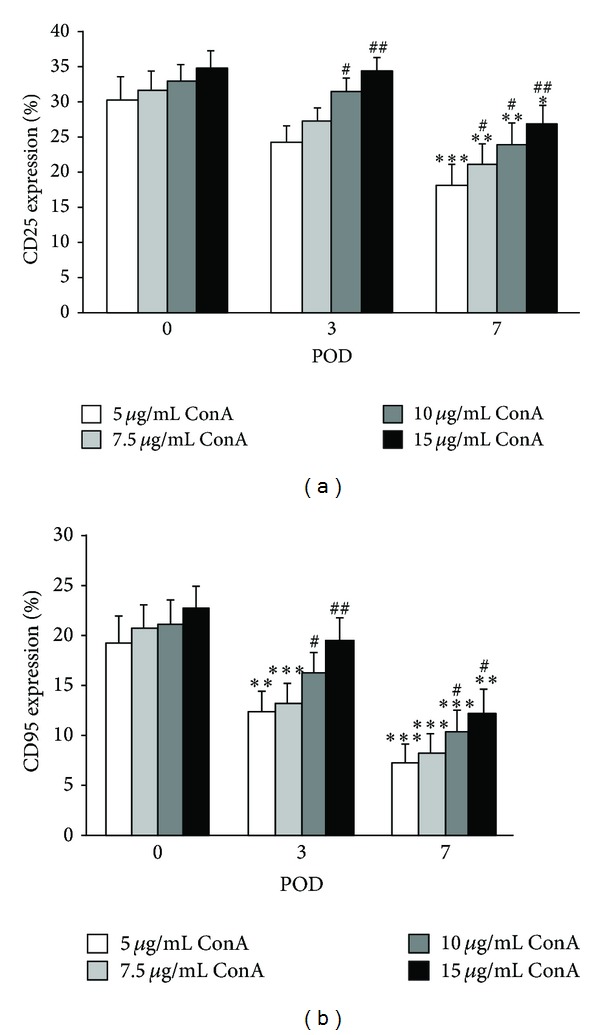
Expression of CD25 (a) and CD95 (b) on whole CD3^+^ T cells population after stimulation with different concentrations of ConA preoperatively (POD-0) and on postoperative days (POD)-3 and -7. Data are represented as mean ± S.E.M. Statistics: Mann-Whitney versus POD-0; *P < 0.02; **P < 0.01; ***P < 0.001. Mann-Whitney versus 5 *μ*g/mL ConA; ^#^ *P* < 0.05; ^##^ *P* < 0.01.

The expression of the activation markers CD25 and CD95 decreased significantly over time with a maximum on POD-7 compared to POD-0 (*P* < 0.02 and *P* < 0.01 for CD25 and CD95, resp.) (Figure 1). There was a poor correlation between mean time on CPBS and the expression of both T cell activation markers (*r* ~ 0.378). Additionally, a significant increase of the expression of CD25 as well as of CD95 after stimulation with rising concentrations of ConA was detected at each time point. Statistically significant differences could be observed on POD-3 (*P* < 0.05 for 10 *μ*g/mL ConA; *P* < 0.02 and *P* < 0.01 for 15 *μ*g/mL ConA, CD25 and CD95, resp.), and POD-7 (*P* < 0.05 for 7.5 *μ*g/mL ConA (CD25); *P* < 0.05 for 10 *μ*g/mL ConA; *P* < 0.01 and *P* < 0.02 for 15 *μ*g/mL ConA, CD25 and CD95, resp.) but not on POD-0.

### 3.3. Expression of T Cell Function Markers in Drug-Treated Whole Blood

**Figure 2:**
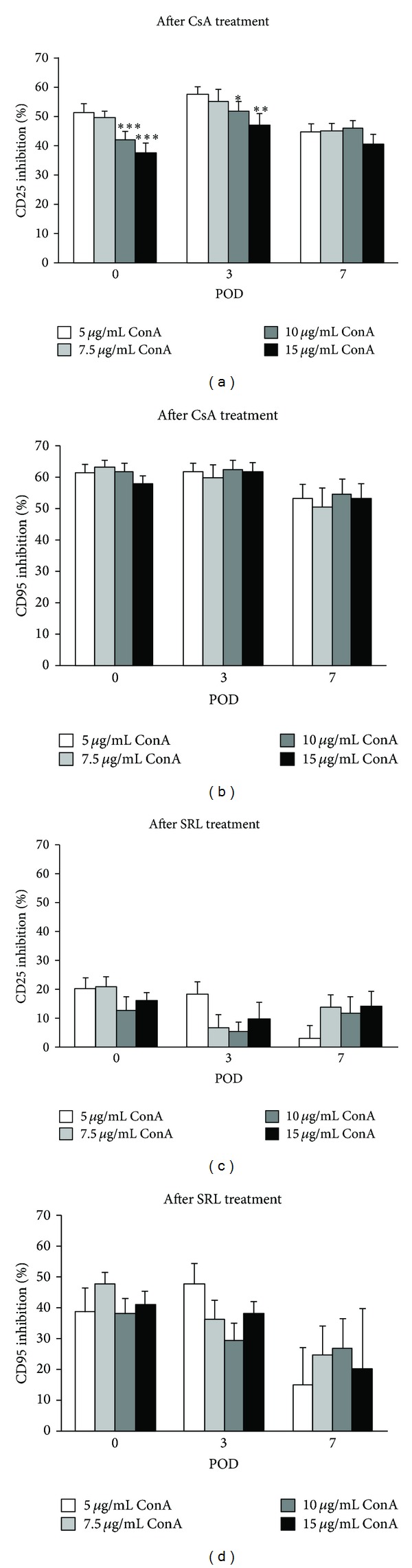
Inhibition of CD25 ((a), (c)) and CD95 ((b), (d)) expression after treatment with Cyclosporin A (CsA; (a), (b)) or sirolimus (SRL; (c), (d)) and stimulation with different concentrations of ConA preoperatively (POD-0) and on postoperative days (POD)-3 and -7. Data are represented as mean ± S.E.M. Statistics: Mann-Whitney versus ConA 5 *μ*g/mL; *P < 0.05; **P < 0.02; ***P < 0.01.

Analysis of T cell function in CsA-treated whole blood showed maximal inhibition of CD25 expression on POD-3, regardless of the ConA stimulation. Again, with increasing ConA stimulation the inhibition of CD25 decreased significantly on POD-0 (*P* < 0.01 5 *μ*g/mL ConA versus 15 *μ*g/mL ConA) and POD-3 (*P* < 0.02 5 *μ*g/mL ConA versus 15 *μ*g/mL ConA), but not on POD-7 (Figure 2(a)). There was a strong inverse correlation between ConA concentration and inhibition of CD25 expression after CsA treatment (POD-0 and POD-3: *r* ~ 0.9; POD-7: *r* ~ 0.7). Inhibition of CD95 expression decreased after CsA treatment only on POD-7 (POD-0 and POD-3 mean expression 61.0% ± 0.01% versus POD-7 52.8% ± 0.03%), being not related to ConA concentrations (Figure 2(b)). There was no correlation between ConA stimulation and inhibition of CD95 expression after CsA treatment, except for POD-0 (*r* ~ 0.81).

After SRL treatment, lowest inhibition of CD25 expression was observed on POD-3. Correlation between ConA concentration and inhibition of CD25 expression on POD-7 was *r* ~ 0.7 (Figure 2(c)). Inhibition of CD95 expression decreased over time, except for a ConA concentration of 5 *μ*g/mL. There was no correlation between ConA concentration and CD95 expression at all study time points (Figure 2(d)). At all time points, inhibition of CD25 and CD95 expression was significantly higher after CsA treatment compared to the inhibition of SRL, regardless the ConA concentration (*P* < 0.001). Additionally, expression of CD25 was less inhibited by immunosuppressive drugs than CD95.

The comparison of all time points revealed that, after addition of SRL, inhibition of antigen expression of both CD25 and CD95 was highest preoperatively, however, after addition of CsA on POD-3, regardless the degree of stimulation (*P* < 0.05).

## 4. Discussion

Impairment of specific parts of the immune system like T cell function after CPBS has been described before and seems to have multifactorial reasons [9]. In this study, we showed for the first time that different pathways of T cell function are affected by CPBS. We analyzed the specific surface markers of T cell function CD25 and CD95 at different time points after CPBS using a flow cytometric whole blood assay.

CD25 is the interleukin-2 receptor *α*-chain, which is expressed in the early phase after T cell activation. The clonal proliferation of activated T cells depends upon the expression of this receptor and resting lymphocytes do not express CD25 [13]. The CD95 (Fas, APO-1) antigen, belonging to the tumor necrosis factor superfamily, is expressed on activated T cells and is involved in proliferation and apoptosis. Interactions of CD95 with its ligand FasL can initiate apoptosis of the target cells, and is one of the mechanisms that cytotoxic T cells use to kill target cells [14]. It has been shown that the quantification of CD95 is a convenient marker for assessing the level of immunosuppression [12–16].

Firstly, we found that expression of CD25 and CD95 significantly decreased over time after CPBS despite increasing absolute lymphocyte counts, indicating that, instead of T cell depletion, different pathways of T cell activation are impaired after CPBS. An increase in lymphocyte counts immediately after surgery has been described before [9]. Another study, however, observed an increase of the neutrophil activation after CPBS [17]. Interestingly, there was no correlation between the time on CPBS and expression of both markers, indicating that not duration but CPBS itself may play an important role in disturbing immune function.

Secondly, we added the immunosuppressive drugs CsA and SRL in specific concentrations to whole blood that are used to prevent rejection after organ transplantation. CsA inhibits the enzyme calcineurin leading to a specific and reversible inhibition of immunocompetent T cells in the G0/G1 phase of the cell cycle, whereas SRL inhibits the *mammalian target of rapamycin* (mTOR), thus leading to a blockade of costimulatory pathways of protein synthesis and cell cycle progression in the G1 phase of T cells [18]. Both immunosuppressive drugs led to inhibition of T cell activation that can be measured by the activation markers CD25 and CD95 [12].

After adding CsA to whole blood, strongest inhibition of both activation markers, CD25 and CD95, was observed on POD-3. Our findings are confirmed by previous results showing a pronounced expression of CD25 on POD-3 [19]. These results might be explained by a special unresponsiveness of the immune system at this time point after CPBS.

In contrast to CsA, the highest inhibition of both antigens after SRL treatment of whole blood was observed preoperatively compared to postoperative values. Our results revealed a change of T cell signaling after CPBS with an altered sensitivity of the signaling for immunosuppressive drugs from the SRL-inhibited pathway preoperatively to a CsA-inhibited pathway in the postoperative course. In general, expression of CD25 was less inhibited by CsA or SRL than the expression of CD95. Furthermore, the inhibition of CD25 and CD95 expression was lower after SRL treatment than after CsA treatment. While CsA is inhibiting calcineurin and therefore blocks the dephosphorylation of the gene-regulating protein NF-AT and consequently the initiation of transcription processes through NF-AT, SRL inhibits several cytokine-induced signaling pathways through complexing mTOR. Both immunosuppressive drugs strongly differ regarding their efficacy on T cell-activating mechanisms and during the postoperative course after CPBS. Based on our results, the inhibition of CD25 and CD95 expression was lower after SRL treatment than after CsA treatment. It might be speculated that in the early course after CPBS calcineurin-dependent pathways are more effected compared to mTOR-dependent pathways of T cell activation. Recent studies confirmed the observation that T cell development was hampered by calcineurin inhibitors like CsA when compared with mTOR-inhibitors like SRL [20, 21].

Our study is limited by the number of patients and the number of time points. Another limitation is the fact that our study was not suited to make any conclusions from the measurements of CD25 to the percentage of regulatory T cells (T_regs_) in patients undergoing CPBS. CD25 is not only expressed by activated T cells, but also by T_regs_. Further studies should therefore include more complex flow cytometric analyses for different types of CD25-positive T cells and subsets of T_regs_ with different immunosuppressive potentials. Flow cytometric analyses should be complemented with measurements of the cell-mediated immunity by the use of the FDA-labeled ImmuKnow-Assay that quantifies the concentration of ATP from CD4 cells following stimulation.

## 5. Conclusion

In this study we demonstrated that different pathways of T cell activation are impaired after CPBS over time. This knowledge may represent the basis to develop interventions that inhibit undesirable effects on T cell activation after CPBS. In addition, further research should investigate the role of different T cell subsets, especially the role of T_regs_ after CPBS.
